# An unusual anatomical variation of the superior turbinate: a case report

**DOI:** 10.1186/1752-1947-8-182

**Published:** 2014-06-09

**Authors:** Turhan San, Emre Gürkan, Bariş Erdoğan

**Affiliations:** 1 Department of ENT, Medeniyet University Göztepe Education and Research Hospital-Turkey, Istanbul, Turkey; 2 Department of ENT, Haydarpaşa Education and Research Hospital-Turkey, Istanbul, Turkey; 3 Department of ENT, Afyonkarahisar Bolvadin State Hospital-Turkey, Istanbul, Turkey

**Keywords:** Complication, Nasal obstruction, Superior turbinate, Variation

## Abstract

**Introduction:**

The only anatomical variation of the superior turbinate defined in the literature is concha bullosa. Determination of anatomical variations of the intranasal structures is important to perform safe endoscopic sinus surgery and avoid complications.

**Case presentation:**

We report a case of an unusual anatomical variation of the superior turbinate in a 55-year-old Turkish man with nasal obstruction and headache.

**Conclusions:**

Anatomical variations of superior turbinate are very rare. Variations of intranasal structures can easily be detected with coronal plane paranasal sinus computed tomography.

## Introduction

Although there are many anatomical variations in the nasal cavity, those related to the superior turbinate are extremely rare. The only variation of the superior turbinate defined in the literature so far is concha bullosa. The determination of anatomical variations in the superior nasal cavity is very important to perform safe endoscopic sinus surgery and avoid complications. Anatomical variations in this region can sometimes cause significant sinonasal symptoms such as nasal obstruction, smell disorders and migraine-like headache. Only a coronal plane paranasal sinus computed tomography (CT) provides detailed information about this inaccessible area of the superior nasal cavity. Therefore, careful evaluation of a paranasal sinus CT scan before surgery is very important in these cases. Here we report a case of unusual anatomical variation of the superior turbinate in a 55-year-old Turkish man.

## Case presentation

**Figure 1:**
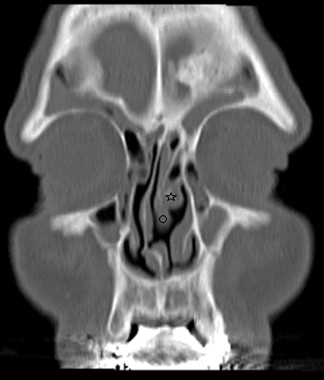
**Coronal paranasal computed tomography slice showing overextension of superior turbinate toward the inferior turbinate on the left and a severe nasal septum deviation on the right.** Also sinusitis in both maxillary sinuses is seen (☆: the middle turbinate, ◯: the superior turbinate).

**Figure 2:**
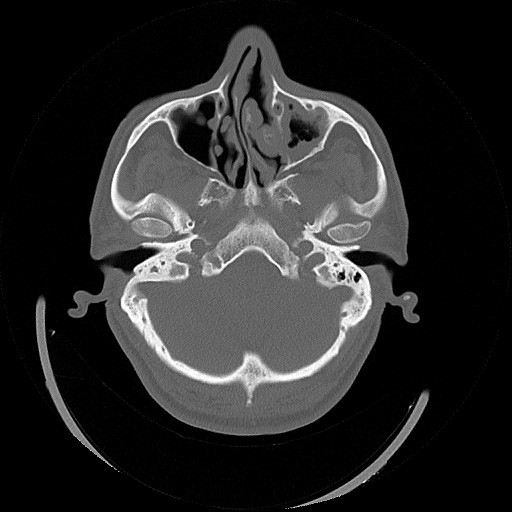
Axial paranasal computed tomography slice showing anatomical variation of superior turbinate.

A 55-year-old Turkish man was admitted to our clinic with complaints of nasal obstruction and headache lasting for years. There was a nasal mass in front of his left middle turbinate extending nearly down to the inferior turbinate in anterior rhinoscopic examination which had an unclear origin. The endoscopic examination revealed a mass extending from his superior nasal cavity toward his inferior turbinate on the left side. The opposite nasal cavity was obstructed by the deviated septum. As shown in Figures 1 and 2, paranasal sinus CT scans showed the overextension of his superior turbinate toward his inferior turbinate on the left side, a severe nasal septum deviation to the right with a spur formation and bilateral inflammatory mucosal thickening in maxillary sinuses. After giving information to the patient about the surgery, resection of the inferior part of the extensive left superior turbinate, minimal invasive endoscopic sinus surgery and septoplasty by endoscopic technique was performed under general anesthesia. He was discharged from the hospital 1 day after the operation. He had an uneventful follow-up period and complete resolution of his complaints. Histologic analysis after surgery revealed turbinate tissue.

## Discussion

Nasal obstruction and headache are common complaints of patients presenting to otorhinolaryngology clinics. Nasal septal deviation and unilateral nasal masses are two common causes of nasal obstruction. The differential diagnosis of unilateral nasal masses which may be congenital, inflammatory, neoplastic or traumatic is important and should be done thoroughly.

The rhinogenic causes of headache are twofold. The first is acute rhinosinusitis and the second is any anatomic variation within the nose. These anatomic variations or anatomical abnormalities can cause headache in and of themselves or as a result of causing sinusitis because of blockage of the osteomeatal complex. These anatomic variations include deviated nasal septum, in particular a spur which may contact either the middle or inferior turbinate (this is the most common cause of rhinogenic headache); congestion of the turbinates; nasal neoplasm; pneumatized agger nasi cells; unusual deflections of uncinated process; paradoxically bent middle turbinate and variations of ethmoid bulla [1]. Pneumatized turbinates have also been reported as a rare cause of headache [2,3]. Any combination of the above or all of the above may cause headache, especially if contact points are noted. Although very rare, sometimes variations of nasal turbinates may also present with similar symptoms. A coronal plane paranasal sinus CT may easily identify such unilateral sinonasal masses and variations of nasal turbinates. Although various variations of the middle turbinate have been defined so far, those related to the superior turbinate are very rare. The superior nasal turbinate has been called the forgotten turbinate because it has been the least accessible and thus the most neglected one among the three turbinates [4].

Changes in turbinate skeletal structure or increased respiratory mucosa volume may constrict the nasal passage causing a negative effect on paranasal sinus ventilation and mucociliary cleaning of the middle meatus which is thought to play a role in the development of sinusitis [2].

Christmas *et al*. [4] and Clerico [5] have suggested that nasal endoscopy does not allow ready access to the superior nasal cavity. A coronal plane paranasal sinus CT can easily determine the anatomic variations of this area and their relations to mucosal pathologies. Endoscopic-limited turbinate resection is the most appropriate treatment option. The most undesirable complications during the surgery are cerebrospinal fluid leak from the anterior skull base and hyposmia as a result of olfactory neuroepithelium damage. To avoid these complications the surgeon must be gentle and make meticulous maneuvers during resection of the superior turbinate. Differential diagnosis on such cases is vital in order to make an accurate and safe treatment. Thus paranasal sinus CT is essential to exclude unilateral anterior skull base pathologies such as nasal encephalocele, meningocele and glioma.

## Conclusions

Anomalies of the superior turbinate are very rare. Paranasal sinus CT can easily identify unusual anatomical variation of the superior turbinate which should be evaluated with great caution by otorhinolaryngologists and radiologists preoperatively.

## Consent

Written informed consent was obtained from the patient for publication of this case report and any accompanying images. A copy of the written consent is available for review by the Editor-in-Chief of this journal.

## Abbreviations

CT: Computed tomography

## Competing interests

The authors declare that they have no competing interests.

## Authors’ contributions

TS made the diagnosis and wrote the case report. EG reviewed and modified. BE supervised. All authors read and approved the final manuscript.
